# Using the Fast Fourier Transform to Accelerate the Computational Search for RNA Conformational Switches

**DOI:** 10.1371/journal.pone.0050506

**Published:** 2012-12-19

**Authors:** Evan Senter, Saad Sheikh, Ivan Dotu, Yann Ponty, Peter Clote

**Affiliations:** 1 Biology Department, Boston College, Chestnut Hill, Massachusetts, United States of America; 2 Computer Science Department, University of Florida, Gainesville, Florida, United States of America; 3 Laboratoire d'Informatique, Ecole Polytechnique, Palaiseau, France; Wake Forest University, United States of America

## Abstract

Using complex roots of unity and the Fast Fourier Transform, we design a new thermodynamics-based algorithm, FFTbor, that computes the Boltzmann probability that secondary structures differ by 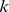 base pairs from an arbitrary initial structure of a given RNA sequence. The algorithm, which runs in quartic time 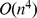 and quadratic space 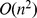, is used to determine the correlation between kinetic folding speed and the *ruggedness* of the energy landscape, and to predict the location of riboswitch expression platform candidates. A web server is available at http://bioinformatics.bc.edu/clotelab/FFTbor/.

## Introduction

In [1], we developed a dynamic programming algorithm, RNAbor, pronounced *RNA neighbor*, which simultaneously computes for each integer 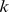, the Boltzmann probability 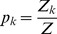 of the subensemble of structures whose base pair distance to a given *initial*, or *reference*, structure 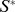 is 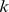. (Here, 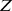 denotes the partition function, defined as the sum of all Boltzmann factors 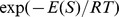, over all secondary structures 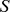 of a given RNA sequence, and 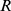 denotes the universal gas constant and 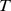 absolute temperature. Similarly 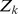 denotes the sum of all Boltzmann factors of all structures 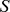, whose base pair distance to the initial structure 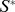 is exactly 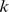.) RNAbor stores the value of the (partial) partition functions 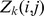 for all 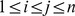 and 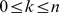, each of which requires quadratic time to compute. Thus it follows that RNAbor runs in time 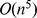 and space 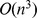, which severely limits its applicability to genomic annotation. This restriction is somewhat mitigated by the fact that in [2], we showed how to use sampling [3] to efficiently approximate RNAbor in cubic time 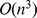 and quadratic space 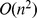, *provided* that the starting structure 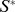 is the minimum free energy (MFE) structure. We expect that a more efficient version of RNAbor could be used in applications in genomics and synthetic biology, to detect potential conformational switches – RNA sequences containing two or more (distinct) metastable structures.

In this paper, we describe a radically different algorithm, FFTbor, prounounced *FFT neighbor*, that uses polynomial interpolation to compute the coefficients 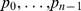 of the polynomial
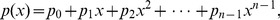
(1)where 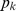 is defined by 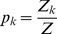. Due to severe numerical instability issues in both the Lagrange interpolation formula and in Gaussian elimination, we employ the Fast Fourier Transform (FFT) to compute the inverse Discrete Fourier Transform (DFT) on values 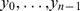, where 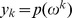 and 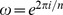 is the principal 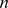
th complex root of unity and 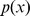 is defined in (1). This gives rise to an improved version of RNAbor, denoted FFTbor, which runs in time 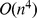 and space 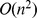. Once two metastable structures 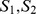 are identified, we can subsequently evaluate the feasibility of transition between structures 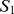 and 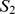, by computing the *barrier energy* using algorithms, such as that described in Dotu et al. [4] or Flamm et al. [5].

### Background

Let 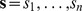 denote an RNA sequence, i.e. a sequence of letters in the alphabet of nucleotides 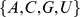. A secondary structure 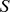 is a set of base pairs 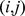, where 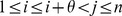 and 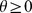 represents the minimum number of unpaired nucleotides in a hairpin loop (due to steric constraints, 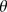 is usually taken to be 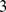), such that if 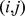 and 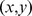 both belong to 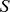, then 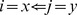 (a nucleotide is involved in at most one base pair) and 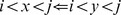 (no pseudoknots are allowed).

The secondary structure 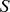 is *compatible* with 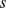 if for every base pair 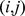 in 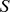, the pair 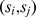 is contained in the set 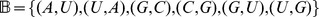 of six Watson-Crick and wobble base pairs. Often we write that 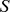 is a secondary structure *on* 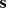, or equivalently, a secondary structure *of* 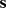, in place of stating that 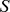 is compatible with 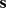. Throughout this paper, by *structure*, we always mean a secondary structure which is compatible with an arbitrary, but fixed RNA sequence 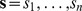.

Given two secondary structures 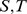 on **s** , we define the base pair distance 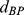 between 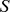 and 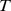 to be the number of base pairs that they have that are not in common, i.e.
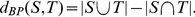
(2)Structures 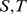 are said to be 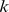
- *neighbors* if 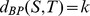.

For 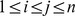, let 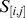 denote the restriction of 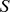 to interval 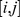 of 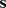, i.e. the set of base pairs 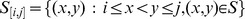. The notion of 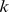-neighbor can also be applied to restrictions of secondary structures; i.e. a secondary structure 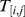 is a 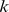 *-neighbor* of 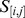 if
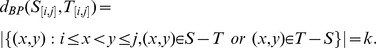
In the following, we often omit the sequence 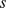 and initial secondary structure 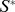 in our notation, since these are arbitrary, but fixed. In particular, we write 
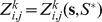
 – see following paragraph for definitions.

**Figure 1:**
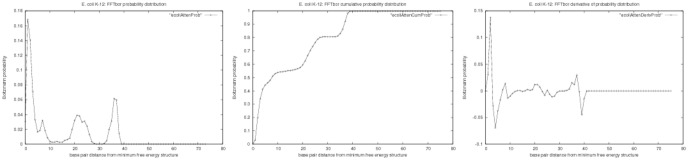
FFTbor output for the RNA attenuator for the phenylalanyl-tRNA synthetase (pheST) operon in *E. coli* K-12 substr. DH10B, located adjacent to the phenylalanyl-tRNA synthetase operon leader, with GenBank accession code CP000948.1/1887748-1887820 (complement). The 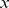-axis represents base pair distance to the minimum free energy structure 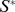; 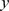-axis represents Boltzmann probability 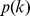 that a structure has base pair distance 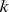 to 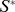. *(Left)* Probability 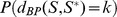 that base pair distance to MFE structure is 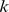. *(Center)* Cumulative probability 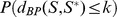 that base pair distance to MFE structure is at most 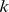. *(Right)* Finite difference (Derivative) 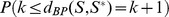 of probability that base pair distance to MFE structure is 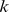.

Given an RNA sequence 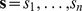 and compatible secondary structure 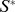, let 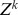 denote the sum of the Boltzmann factors 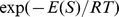 of all 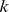-neighbors 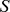 of 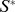; i.e.
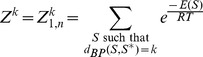
where 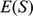 denotes the Turner (nearest neighbor) energy [6], [7] of 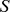, 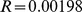 kcal/mol denotes the universal gas constant and 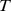 denotes absolute temperature. Since the maximum base pair distance between a given initial structure 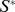 and any other structure 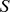 on RNA sequence 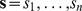 must satisfy
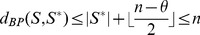
(3)it follows that the full partition function
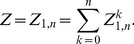
(4)Moreover, since 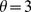, we need to compute at most the values 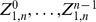 – this observation will later prove useful. The Boltzmann probability 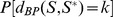 that a secondary structure 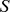 has base pair distance 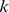 from the initial structure 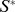 can be defined from the partition function by
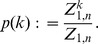
By graphing the probabilities 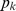 as a function of 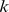, we expect to see one or more peaks at base pair distance 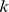 when there is a meta-stable (low energy) structure 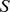 at base pair distance 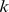 from 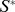. See Figure 1 for an illustration.

### Recursions for structural neighbors

For the rest of the paper, we consider both 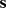 as well as the secondary structure 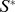 on 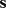 to be fixed. We now recall the recursions from Freyhult et al. [8] to determine the partition function 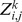 with respect to the Nussinov-Jacobson energy 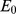 model [9], defined by 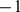 times the number of base pairs; i.e. 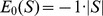. Although we describe here the recursions for the Nussinov-Jacobson model, for the sake of simplicity of exposition, both RNAbor [8] as well as our current software FFTbor, concern the Turner energy model, consisting of free energy parameters for stacked bases, hairpins, bulges, internal loops and multiloops. The full recursions for FFTbor are described for the the Turner energy model in the appendix.

The base case for 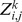 is given by
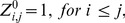
(5)since the only 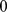-neighbor to a structure 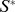 is the structure 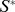 itself, and
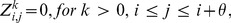
(6)since the empty structure is the only possible structure for a sequence shorter than 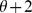 nucleotides, and so there are no 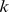-neighbors for 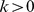. The recursion used to compute 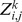 for 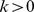 and 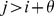 is
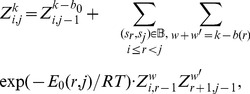
(7)where 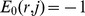 if positions 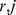 can pair in sequence 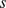, and otherwise 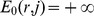. Additionally, 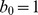 if 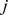 is base-paired in 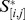 and 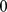 otherwise, and 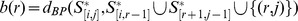. This holds since in a secondary structure 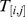 on 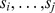 that is a 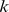-neighbor of 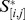, either nucleotide 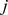 is unpaired in 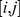 or it is paired to a nucleotide 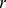 such that 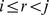. In this latter case it is enough to study the smaller sequence segments 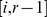 and 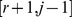 noting that, except for 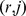, base pairs outside of these regions are not allowed, since there are no pseudoknots. In addition, for 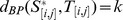 to hold, it is necessary for 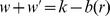 to hold, where 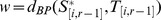 and 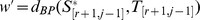, since 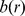 is the number of base pairs that differ between 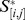 and a structure 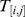, due to the introduction of the base pair 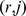.

## Methods

Given RNA sequence 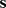 and compatible initial structure 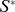, we define the *polynomial*

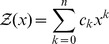
(8)where coefficients 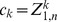. Moreover, because of (3) and the fact that the minimum number of unpaired bases in a hairpin loop 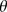 is 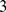, we know that 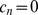, so that 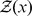 is a polynomial of degree strictly less than 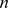. If we evaluate the polynomial 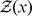 for 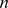 distinct values
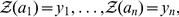
(9)then the Lagrange polynomial interpolation formula guarantees that 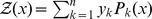, where the polynomials 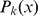 have degree at most 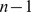 and are given by the Lagrange formula
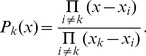
(10)Since the polynomials 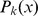 can be explicitly computed, it follows that we can compute the coefficients 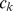 of polynomial 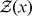. As we describe below, the evaluation of 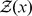 for a fixed value of 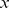 can be done in time 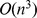 and space 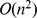. It follows that the coefficients 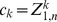 can be computed after 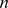 evaluations of 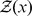, where the space for each evaluation of 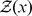 is re-used; hence these evaluations can be performed in time 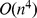 and space 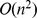. Finally, Lagrange interpolation is clearly computable in time 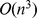. Although this approach is theoretically sound, there are severe numerical stability issues related to the interpolation method [10], the choice of values 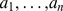 in the interpolation, and floating point arithmetic (round-off error) related to the astronomically large values of the partition functions 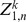, for 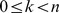. After many unsuccessful approaches including scaling (see File S1), we obtained excellent results by interpolating the polynomial 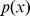, defined in equation (1), rather than the polynomial 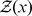, defined in equation (9), and performing interpolation with the Fast Fourier Transform (FFT) [11] where 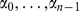 are chosen to be 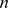
th complex roots of unity, 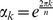. One advantage of the FFT is that interpolation can be performed in 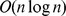 time, rather than the cubic time required by using the Lagrange formula (10) or by Gaussian elimination. Fewer numerical operations implies increased numerical stability in our application. Details now follow.

### Recursions to compute the polynomial 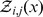


Given an initial secondary structure 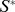 of a given RNA sequence 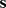, our goal is to compute
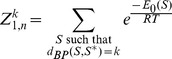
(11)where 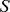 can be any structure compatible with 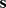. As previously mentioned, the recurrence relation for RNAbor with respect to the Nussinov energy model 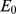 is
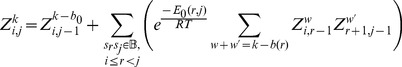
(12)where 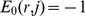 if 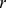 and 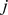 can base-pair and otherwise 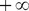, and 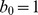 if 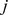 is base paired in 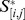 and 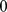 otherwise, and 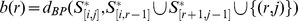. The following theorem shows that an analogous recursion can be used to compute the *polynomial* 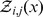 defined by
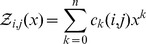
(13)where
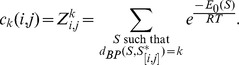
Here, in the summation, 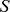 runs over structures on 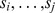, which are 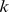-neighbors of the restriction 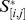 of initial structure 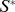 to interval 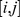, and 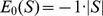 denotes the Nussinov-Jacobson energy of 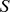.

Theorem 1: Let 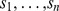 be a given RNA sequence. For any integers 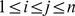, let
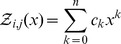
where
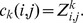
Then for 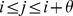, 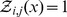 and for 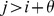 we have the recurrence relation
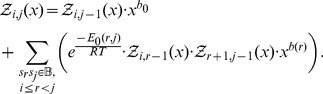
(14)where 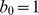 if 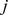 is base-paired in 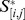 and 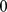 otherwise, and 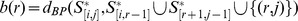.

Proof: First, some notation is necessary. Recall that if 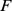 is an arbitrary polynomial [resp. analytic] function, then 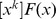 denotes the coefficient of 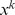 [resp. the *k*th Taylor coefficient in the Taylor expansion of 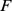] – for instance, in equation (1), 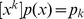, and in equation (9), 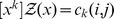.

By definition, it is clear that 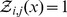 if 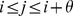, where we recall that 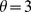 is the minimum number of unpaired bases in a hairpin loop. For 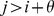, we have
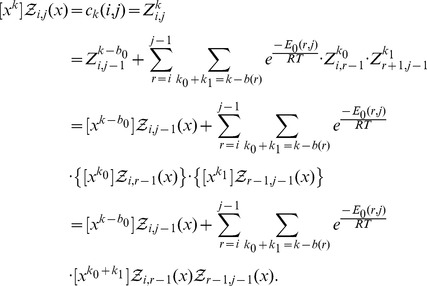
By induction, the proof of the theorem now follows. 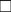


Notice that if one were to compute all terms of the polynomial 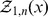 by explicitly performing polynomial multiplications, then the computation would require 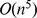 time and 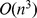 space. Instead of explicitly performing polynomial expansion in *variable* 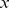, we instantiate 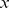 to a fixed complex number 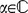, and apply the following recursion for this instantiation:
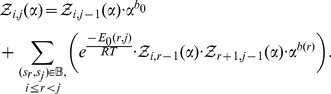
(15)In this fashion, we can compute 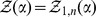 in 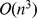 time and 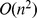 space. For 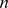 distinct complex values 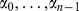, we can compute and save only the values 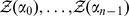, each time re-using the 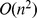 space for the next computation of 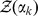. It follows that the computation resources used to determine the (column) vector
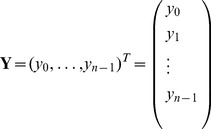
(16)where 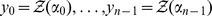 is thus quartic time 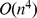 and quadratic space 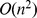.

### Polynomial interpolation using the FFT

Let 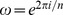 be the principal 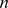
th complex root of unity. Recall that the Vandermonde matrix 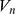 is defined to be the 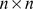 matrix, whose 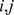 entry is 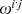; i.e.
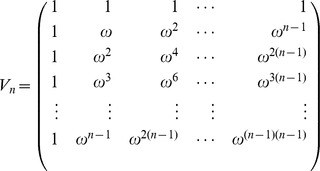
The Fast Fourier Transform (FFT) is defined to be the 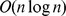 algorithm to compute the Discrete Fourier Transform (DFT), defined as the matrix product 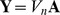:
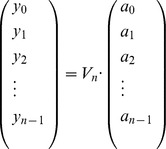
On page 837 of [11], it is shown that the 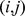 entry of 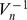 is 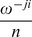 and that
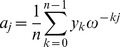
(17)for 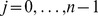.

Since we defined 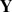 in (16) by 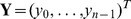, where 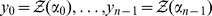 and 
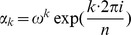
, it follows that the coefficients 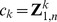 in the polynomial 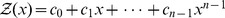 defined in (8) can be computed, at least in principle, by using the FFT. It turns out, however, that the values of 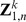 are so astronomically large, that the ensuing numerical instability makes even this approach infeasible for values of 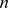 that exceed 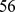 (data not shown). Nevertheless, our approach can be modified as follows. Define 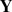 by 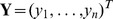, where 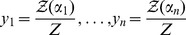, and 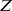 is the partition function defined in (4). Using the FFT to compute the inverse DFT, it follows from (17) that we can compute the probabilities 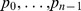 that are coefficients of the polynomial 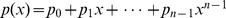 defined in equation (1). For genomics applications, we are only interested in the 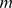 most significant digits of each 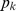, as described in the pseudocode below.

Algorithm for FFTbor

This pseudocode computes the 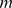 most significant digits of probabilities 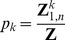.

 Input: RNA sequence 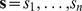, and initial secondary structure 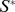 of 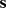, and integer 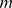.

Output: Probabilities 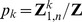 to 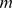 significant digits for 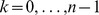.

1. generate roots of unity 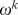 for 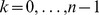, where 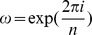 and 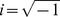

2. note that the partition function 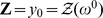

3. for 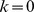 to 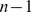

4.  compute 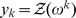 using recursion (15)
 

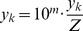
 //normalize 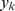

6. compute 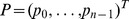 where 
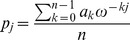
 by using FFT in (17)
7. for 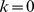 to 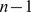

 

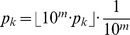

9.  //truncate to 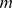 most significant digits

#### Speed-up in our implementation of FFTbor

In this subsection, we show that we need only evaluate the polynomial 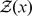, as defined in equation (8), for 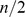 of the complex 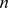
th roots of unity. It is first necessary to recall the definition of complex conjugate. Recall that the complex conjugate of 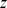 is denoted by 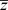; i.e. if 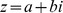 where 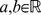 are real numbers and 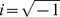, then 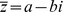.

Lemma 1: If 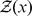 is the complex polynomial defined in equation (8), then for any complex 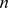
th root of unity 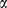, it is the case that 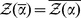. In other words, if 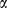 is a complex 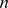
th root of unity of the form 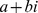, where 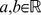 and 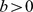, and if 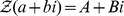 where 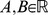, then it is the case that
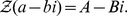



Proof: Letting 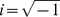, if 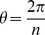, then 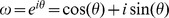 is the principal 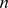
th complex root of unity, and 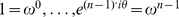 together constitute the complete collection of all 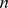
th complex roots of unity – i.e. the 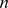 unique solutions of of the equation 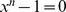 over the field 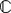 of complex numbers. Clearly, for any 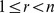, 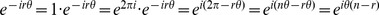. Moreover, if 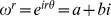 where 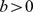, then we have 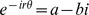. It follows that for any 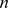
th root of unity of the form 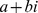, where 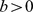, the number 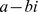 is also an 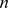
th root of unity.

Recall that 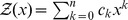, where 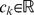 are real numbers representing the partition function 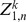 over all secondary structures of a given RNA sequence 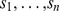, whose base pair distance from initial structure 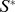 is 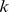. Thus, in order to prove the lemma, it suffices to show that for all values 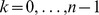, if 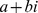 is a complex 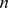
th root of unity, where 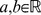 and 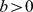, and if 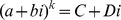 where 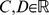, *then* 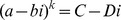. Indeed, we have the following.
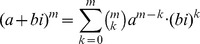


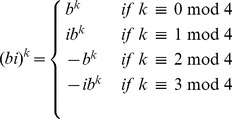


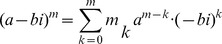


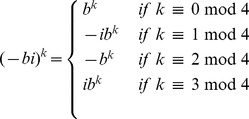
It follows that each term of the form 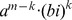, for 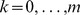, is the complex conjugate of 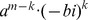, and thus 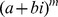 is the complex conjugate of 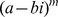. Since 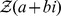 is a sum of terms of the form 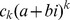, it follows that 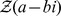 is the complex conjugate of 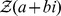. This completes the proof of the lemma. 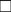


Lemma 1 immediately entails that we need only evaluate 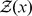 on 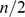 many of the complex 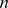
th roots of unity – namely, those of the form 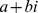, where 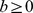. The remaining values of 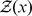 are obtained by taking conplex conjugates of the first 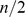 values. This, along with a precomputation of powers of the complex 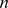
th roots of unity, leads to an enormous performance speed-up in our implementation of FFTbor.

## Results

### Applications of FFTbor

In this section, we consider two applications of FFTbor: *(i)* correlation between kinetic folding speed and the *ruggedness* of the energy landscape near the minimum free energy structure, *(ii)* computational detection of riboswitch expression platform candidates.

#### Kinetic folding speed and energy landscape ruggedness

**Figure 2:**
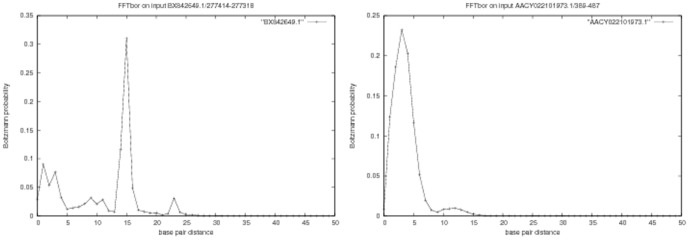
Output from FFTbor on two randomly selected thiamine pyrophosphate riboswitch (TPP) aptamers, taken from the Rfam database [**23**] **.** The 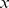-axis represents base pair distance from the minimum free energy structure for each given sequence; the 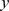-axis represents Boltzmann probabilities 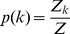, where 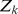 denotes the sum of Boltzmann factors or all secondary structures, whose base pair distance from the MFE structure is exactly 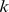. *(Left)* The 97 nt sequence BX842649.1/277414-277318 appears to have a rugged energy landscape near its minimum free energy structure, with distinct low energy structures that may compete with the MFE structure during the folding process. *(Right)* The 99 nt sequence, AACY022101973.1/389-487 appears to have a smooth energy landscape near its MFE structure, with no distinct low energy structures to might compete with the MFE structure. Based on the FFTbor output or *structural profile* near MFE structure 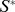, one might expect folding time for the first sequence to increase due to competition from metastable structures, while one might expect the second sequence to have rapid folding time. Computational Monte Carlo folding experiments bear out this fact. Kinfold [15] simulations clearly show that the second sequence folds at least four times more quickly than the first sequence. See text for details. Subfigure A Subfigure B Subfigure C Subfigure D.

The output of FFTbor, as shown in Figure 2, is a probability distribution, where the 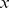-axis represents the base pair distance from an arbitrary, but fixed secondary structure 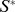, and the 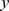-axis represents the Boltzmann probability 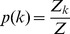 that a secondary structure has base pair distance 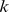 from 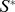. Arguably, this probability distribution is an accurate one-dimensional projection of the rugged, high dimensional energy landscape near structure 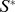, of the sort artistically rendered in the well-known energy landscape depicted in Figure 1 of [12]. In the sequel, we may call the FFTbor probability distribution a *structural neighbor profile*, or simply *structural profile* 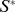. A hypothesis behind theoretical work in biomolecular folding theory in [13] is that kinetic folding slows down as the energy landscape becomes more *rugged*. This is borne out in our computational experiments for RNA using FFTbor, as reported in Figure 2.

**Figure 3:**
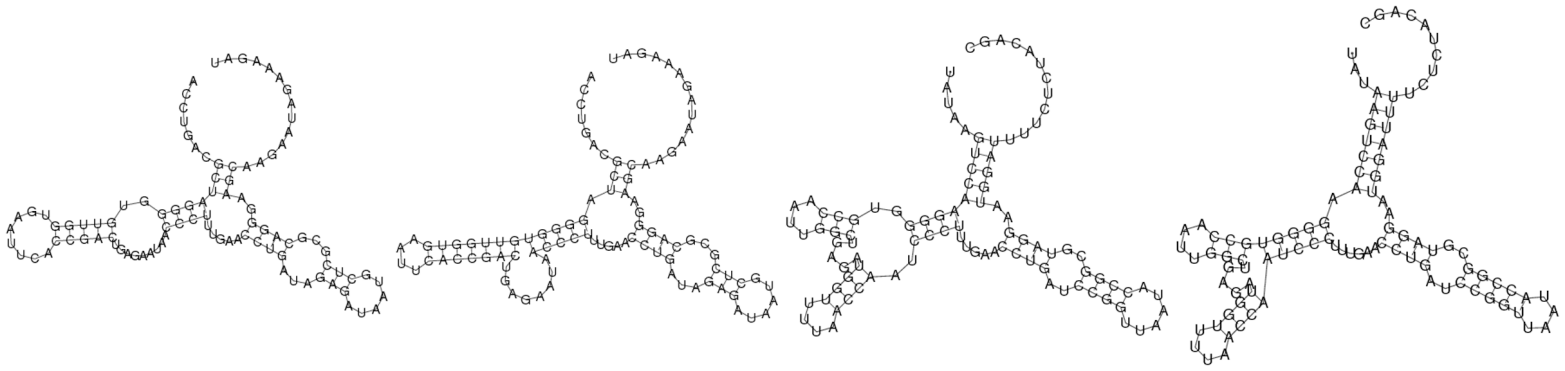
Rfam consensus structures (Rfam) and minimum free energy (MFE) secondary structures for two thiamine pyrophosphate (TPP) riboswitch aptamers, chosen at random from RF00059 Rfam family seed alignment [**23**]. Using pairwise BLAST [14], there is no sequence similarity, although the secondary structures are very similar, as shown in this figure. *(A)* Rfam consensus structure for BX842649.1/277414-277318. *(B)* MFE structure for BX842649.1/277414-277318. *(C)* Rfam consensus structure for AACY022101973.1/389-487. *(D)* Rfam consensus structure for AACY022101973.1/389-487.

We randomly chose two TPP riboswitch aptamers from the seed alignment for Rfam family RF00059. The first sequence has EMBL accession code BX842649.1/277414-277318 and is comprised of the 97 nt sequence ACCUGACGCU AGGGGUGUUG GUGAAUUCAC CGACUGAGAA UAACCCUUUG AACCUGAUAG AGAUAAUGCU CGCGCAGGGA AGCAAGAAUA GAAAGAU, while the second sequence has EMBL accession code AACY022101973.1/389-487 and is comprised of the 99 nt sequence UAUAAGUCCA AGGGGUGCCA AUUGGCUGAG AUGGUUUUAA CCAAUCCCUU UGAACCUGAU CCGGUUAAUA CCGGCGUAGG AAUGGAUUUU CUCUACAGC. Rfam consensus and minimum free energy structures for both sequences are depicted in Figure 3. Despite the fact that there is no sequence homology according to pairwise BLAST [14], this figure clearly demonstrates that consensus and minimum free energy structures closely resemble each other, and that the structures of both TPP riboswitch aptamers are quite similar, with the exception of the leftmost hairpin loop [resp. multiloop]. The MFE structures differ from the consensus structures principally by the addition of base pairs not determined by covariation in the Rfam alignment. Indeed, if we let 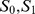 denote the Rfam consensus structure resp. MFE structure for the 97 nt sequence with EMBL accession code BX842649.1/277414-277318, then 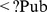 has 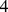 base pairs, and 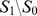 has 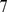 base pairs. If we let 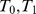 denote the Rfam consensus structure resp. MFE structure for the 99 nt sequence with EMBL accession code AACY022101973.1/389-487, then 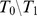 has 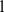 base pair, and 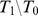 has 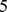 base pairs.

We ran FFTbor on each of the TPP riboswitch aptamer sequences, with the MFE structure of each sequence taken as the initial structure 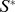 for that sequence. For the first sequence, BX842649.1/277414-277318, the FFTbor output suggests that there are low energy structures at a distance from the MFE structure, which might compete with the MFE structure and hence slow the kinetics of folding. In contrast, for the second sequence, AACY022101973.1/389-487, the FFTbor output suggests that there are no such competing low energy structures, hence the second sequence should fold more quickly than the first.

To test the hypothesis that folding is slower for rugged energy landscapes, we ran the kinetic folding software, Kinfold [15], on each of the two TPP riboswitch aptamer sequences, BX842649.1/277414-277318 and AACY022101973.1/389-487, to determine the *mean first passage time* (MFPT) to fold into the MFE structure, when starting from the empty structure. In this computational experiment, we took MFPT to be the average number of Monte Carlo steps taken by Kinfold, each step consisting of the addition or removal of a single base pair (or shift – see [15]), to fold the empty structure into the MFE structure, where the average was taken over 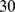 runs, with an absolute maximum number of Monte Carlo steps taken to be 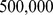. The first sequence, BX842649.1/277414-277318, converged within 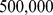 steps only for 20 out of 30 runs. Assigning the maximum step count of 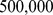 for the 10 runs that did not converge, we found a mean first passage time of 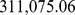 steps for this sequence. The second sequence, AACY022101973.1/389-487, converged within 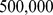 steps in 29 out of 30 runs, and we found a mean first passage time of 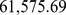 steps for this sequence. From computational experiments of this type, it is suggestive that FFTbor may prove useful in synthetic biology, where one would like to design rapidly folding RNA molecules that fold into a designated target structure. (See [16], [17], [18], [19] for more on synthetic biology.) In particular, one could use RNAinverse [20], RNA-SSD [21], INFO-RNA [22], or our recent constraint programming exact solution of RNA inverse folding, RNAiFold (to appear in Journal of Bioinformatics and Computational Biology, see http://bioinformatics.bc.edu/clotelab/RNAiFold/), to output a list of sequences, whose minimum free energy structure is a designated target structure. Subsequently, using FFTbor, one could prioritize sequences in terms of FFTbor structural profile, on the grounds that sequences with a profile similar to the right panel of Figure 2 are likely to fold more rapidly than those whose profile resembles the left panel of Figure 2.

In order to more systematically determine the relation between kinetic folding speed and the ruggedness of an energy landscape near the MFE structure, we need to numerically quantify ruggedness. To this end, in the following we define the notion of *expected base pair distance* to a designated structure. Let 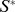 be an arbitrary secondary structure of the RNA sequence 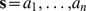. The expected base pair distance to 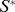 is defined by
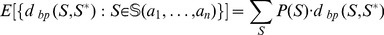
(18)where 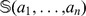 denotes the set of secondary structures for 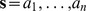, 
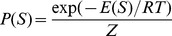
 is the Boltzmann probability of 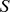, and 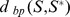 denotes base pair distance between 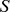 and 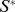. If we run FFTbor on an input sequence 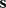 and secondary structure 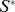, then clearly 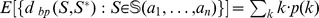, where 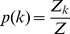, obtained from the program output. If 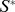 is the empty structure, then FFTbor output is simply the probability distribution of the number of base pairs per secondary structure, taken over the Boltzmann ensemble of all structures.

**Figure 4:**
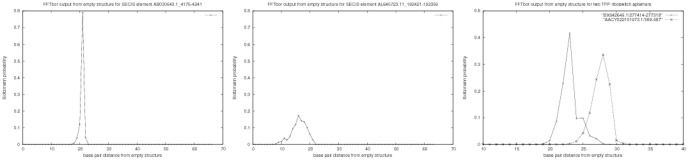
This figure represents the graphical output of FFTbor, when the empty structure is chosen as initial structure 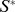
 **.** The 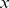-axis represents the number of base pairs per structure, taken over the ensemble of all secondary structures for the given RNA sequence; the 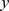-axis represents Boltzmann probability 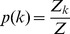, where 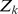 is the partition function for all secondary structures having exactly 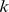 base pairs. *(Left)* For the selenocysteine (SECIS) element AB030643.1/4176-4241 from Rfam family RF00031, the standard deviation 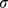 of the number of base pairs, taken over the ensemble of all secondary structures, is 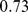, while the logarithm base 10 of the mean first passage time (logMFPT) is 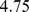. *(Center)* For the selenocysteine (SECIS) element AL645723.11/192421-192359 from Rfam family RF00031, the standard deviation 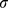 of the number of base pairs, taken over the ensemble of all secondary structures, is 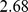, while logMFPT is 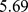. Among the 61 sequences in the seed alignment of RF00031, AB030643.1/4176-4241 was the fastest folder, while AL645723.11/192421-192359 was the slowest folder. *(Right)* Superimposition of output of FFTbor for two TPP riboswitch aptamers: the 97 nt sequence BX842649.1/277414-277318 and the 99 nt sequence AACY022101973.1/389-487, both obtained when taking the empty structure for the initial structure 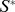. The mean 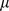 for the FFTbor structural profile near the empty structure is 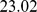 [resp. 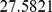], the standard deviation 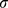 for the FFTbor structural profile is 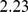 [resp. 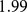], and the Kinfold MFPT is 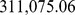 [resp. 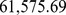] for the TPP riboswitch aptamer AB030643.1/4176-4241 [resp. AL645723.11/192421-192359]. The right panel of this figure should be compared with Figure 2. These anecdotal results bear up the correlation between standard deviation 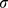 and logMFPT described in Table 1.

For the benchmarking assay, we took all 61 selenocysteine insertion sequence (SECIS) sequences from the seed alignment of Rfam family RF00031 [23]. Average length was 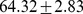 nt. For each sequence, we ran both FFTbor and a Monte Carlo folding algorithm, developed by E. Freyhult and P. Clote (unpublished). Using the Monte Carlo algorithm, we determined the mean first passage time (MFPT), defined as the average taken over 50 runs, of the number of Monte Carlo steps taken to fold the empty structure into the MFE structure, where an absolute upper bound of 5 million steps was allowed in the simulation. After unsuccessful attempts due to *ruggedness* of the energy landscape near the MFE structure, by using the Hartigan-Hartigan *dip* test of unimodality [24], expected base pair distance from MFE structure, total variation distance between FFTbor output and the exponential distribution estimated by the method of moments [25], etc., we ran FFTbor when starting from the empty structure (rather than the MFE structure) as initial structure. As mentioned above, in this case, FFTbor output is simply the probability distribution for the number of base pairs per structure, taken over the ensemble of all secondary structure for the input RNA sequence. Surprisingly, we found that there is a significant correlation of 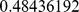 with one-tailed 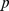-value of 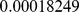 between the standard deviation of the FFTbor output (when starting from the empty structure) and logarithm base 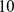 of the mean first passage time. Table 1 and Figure 4 explain this phenomenon in detail.

**Table 1:** Pearson correlation between various aspects of selenocysteine insertion sequences from the seed alignment of Rfam family RF00031 [23].

	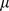	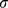	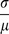	len	MFE	logMFPT
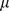	1					
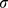	−0.43722448	1				
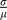	−0.691411183	0.943650913	1			
len	0.707683898	−0.158951202	−0.364591789	1		
MFE	−0.569474125	0.739515083	0.759622716	−0.368485646	1	
logMFPT	−0.036291124	0.48436192	0.376230235	0.405865529	0.399015556	1

For each of the 61 RNA sequences, we ran FFTbor, starting from empty initial structure 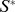, and we ran a Monte Carlo folding algorithm, developed by E. Freyhult and P. Clote (unpublished). Using the Monte Carlo algorithm, we determined the mean first passage time (MFPT), defined as the average taken over 50 runs, of the number of Monte Carlo steps taken to fold the empty structure into the MFE structure, where an absolute upper bound of 5 million steps was allowed in the simulation. From the output of FFTbor, we computed *(1)* the mean number (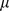) of base pairs per structure, taken over the ensemble of all secondary structures for the given sequence, *(2)* the standard deviation (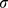) of the number of base pairs per structure, *(3)* the coefficient of variation 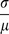, *(4)* the RNA sequence length, and *(5)* the minimum free energy (MFE). Additionally, we computed the logarithm base 10 of mean first passage time (log10MFPT), taken over 50 Monte Carlo runs per sequence (log base 10 of the standard deviation of number of Monte Carlo steps per run was approximately 9% of log10MFPT on average). The table shows the correlation between each of these aspects. Some correlations are obvious – for example, *(i)* the standard deviation 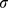 is highly correlated with the coefficient of variation 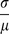; *(ii)* the mean 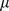 is negatively correlated with the coefficient of variation 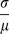; *(iii)* the mean 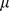 is negatively correlated with the minimum free energy (MFE) – if most low energy structures in the ensemble have many base pairs, then it is likely that the minimum free energy is very low (i.e. since MFE is negative, the absolute value of MFE increases); *(iv)* sequence length is negatively correlated with MFE – as sequence length increases, the minimum free energy (MFE) decreases. However, it may appear surprising that *(v)* the mean 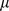 number of base pairs per structure is independent of MFPT (correlation 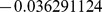), although *(vi)* MFE is correlated with MFPT (correlation 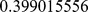) – i.e. from *(iii)*, lower MFE is correlated with a larger average 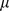 number of base pairs per structure, from *(vi)* higher MFE is correlated with longer folding time, but from *(v)* the average 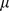 number of base pairs per structure is independent of folding time. The most important insight from this table is that *(vii)* standard deviation 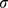 is correlated with mean first passage time – the correlation is statistically significant, with one-tailed 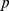-value of 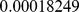.

In the right panel of Figure 4, we applied FFTbor to each of the two randomly chosen TPP riboswitch aptamers BX842649.1/277414-277318 and AACY022101973.1/389-487, starting from the empty reference structure 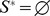. The mean for the FFTbor structural profile near the empty structure is 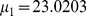 [resp. 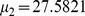], the standard deviation 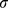 for the FFTbor structural profile is 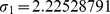 [resp. 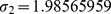], and the Kinfold MFPT is 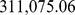 [resp. 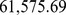] for the TPP riboswitch aptamer AB030643.1/4176-4241 [resp. AL645723.11/192421-192359]. This anecdotal evidence supports the hypothesis that small standard deviation in FFTbor distribution is correlated with fast folding.

Additionally, in following a suggestion of one of the anonymous referees, we randomized the TPP riboswitches BX842649.1/277414-277318 and AACY022101973.1/389-487 by using our implementation of the Altschul-Erikson dinucleotide shuffle algorithm [26], and then applied FFTbor to these sequences, starting from the empty structure. The mean 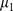 and standard deviation 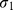 for the FFTbor distribution for randomized BX842649 are respectively 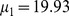 and 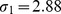, while those for randomized AACY022101973 are 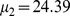 and 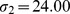. Running Kinfold, with a maximum of 500,000 steps with 30 replicates (as explained in the text), we found that for randomized BX842649, all 30 runs converged yielding a mean first passage time (MFPT) of 13022.58 with standard deviation of 15221.78. In contrast for randomized AACY022101973, only 15 out of 30 runs converged within 500,000 steps, and discounting these nonconvergent data, we obtain an average mean first passage time (MFPT) of 94446.93 with standard deviation of 157107.43. This additional test provides more anecdotal evidence supporting our hypothesis that small standard deviation 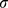 in FFTbor probability density is correlated with fast folding, as measured by MFPT.

#### Riboswitch expression platform prediction

A bacterial riboswitch is a portion of the 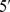 untranslated region (UTR) of messenger RNA, that performs gene regulation by undergoing a conformational change upon binding with a ligand, such as guanine, thiamine pyrophosphate, lysine, etc. [27]. This conformational change may either turn on or off the corresponding gene by either transcriptional or translational regulation of the messenger RNA [28], depending on the particular riboswitch. The common feature shared by all riboswitches is that a gene is regulated by conformational change upon ligand binding. Bacterial riboswitches are often found upstream of operons, regulating groups of genes, as in purine *de novo* synthesis and salvage [29].

A riboswitch consists of two equally important parts: an upstream *aptamer*, capable of highly discriminative binding to a particular ligand, and a downstream *expression platform*, capable of undergoing a radical conformational change upon binding of a ligand with the discriminating aptamer. Since aptamers have been under strong evolutionary pressure to bind with high affinity (e.g. 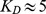 nM for guanine [30]), there is strong sequence conservation found in the aptameric region of orthologous riboswitches. In contrast, while secondary structure is conserved in the terminator loop of the expression platform in purine riboswitches, there is relatively low sequence conservation (data not shown). While a number of methods exist to computationally predict riboswitch aptamers [31], [32], [33], [34], [35] (and especially INFERNAL [36], which latter is used to predict riboswitch aptamers in Rfam), it is an important biological problem to determine the expression platform, since the structure of the expression platform can suggest whether there is transcriptional regulation via a terminator loop or translational regulation via the sequestration of the Shine-Dalgarno sequence [28]. Determination of the precise location and structure of the expression platform is difficult due to low conserved sequence identity (in-house computations, data not shown). Although this problem remains open, we report here how FFTbor may provide help to biologists in the selection and prioritization of riboswitch candidates.

**Figure 5:**
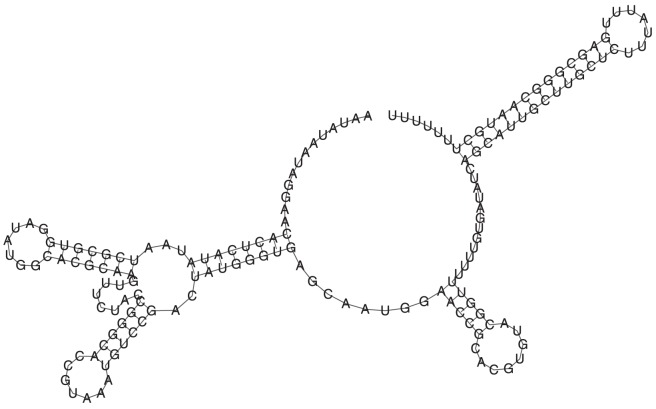
Gene OFF secondary structure of the xpt G-box purine riboswitch in *B. subtilis*; structure taken from that in **Figure 1A** of [**27**].

**Figure 6:**
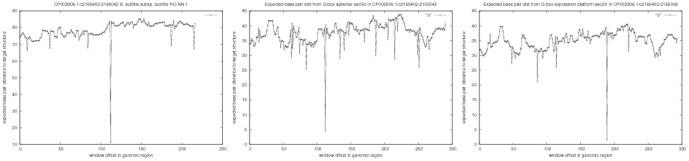
Graph of the expected distance from target secondary structure, as a function of window offset position in the 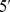
 **untranslated region (UTR) of the xpt gene of** ***B. subtilis*** **; i.e. GenBank accession code CP002906.1/c2165402-2165042** ***B. subtilis*** **subsp. subtilis RO-NN-1.** In a moving window application, FFTbor computed the Boltzmann probability 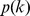 that secondary structures of the current window contents have base pair distance 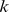 from the target (or initial) structure 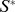. In each case, the size of the window was set to equal the length of 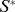. *(Left)* Target structure 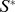 comprises the entire secondary of the xpt riboswitch, as depicted in Figure 5, with the exception that the leading and trailing unpaired positions were removed, as explained in the text – see displayed dot bracket structure in (19). *(Center)* Target structure 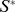 comprises only the aptamer secondary structure, as displayed in dot bracket structure in (20). *(Right)* Target structure 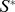 comprises only the expression platform secondary structure, as displayed in dot bracket structure in (21). The number of points displayed on the 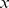-axis differs in each case, since the window size differs, as explained above. The very well-defined minimum in each panel corresponds to the exact location of the entire riboswitch (left panel), aptamer (center panel) and expression platform (right panel). Note that the base line value for the expected base pair distance in the left panel (entire riboswitch) is approximately 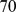, while that for both the center panel (aptamer) and right panel (expression platform) is approximately 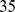.

Figure 5 depicts the gene OFF structure of the xpt G-box purine riboswitch in *B. subtilis*, as determined by inline-probing – this structure was taken from Figure 1 of [27]. Note that this structure is only partial, since there are regions with no base pairs depicted, despite the fact that additional base pairs could be added. By using blastn, it is found that this 161 nt purine riboswitch can be found on the complement strand of GenBank accession number CP002906.1/c2165302-2165142 in complete genome of *B. subtilis subsp. subtilis RO-NN-1*. Figure 6 depicts the result of three computational experiments with FFTbor. The left panel displays the expected base pair distance to the following secondary structure
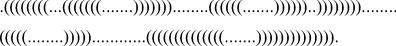
(19)as a function of window offset, where window size equals the size of this target structure. This structure was obtained by removing all leading and trailing unpaired positions from the structure depicted in Figure 5, except for the leftmost [resp. rightmost] unpaired position adjacent to the leftmost [resp. rightmost] base-paired position. The reason for removal of the leading and trailing unpaired positions was that the structure of [27], depicted in Figure 5, is clearly only partial, as earlier mentioned. The center panel displays the expected base pair distance to the following secondary structure
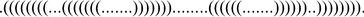
(20)as a function of window offset, where window size equals the size of this target *aptamer* structure. Similarly, the right panel displays the expected base pair distance to the following secondary structure
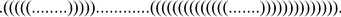
(21)as a function of window offset, where window size equals the size of this target *expression platform* structure. Figure 6 determines the precise location of the xpt riboswitch, both aptamer and expression platform.

**Figure 7:**
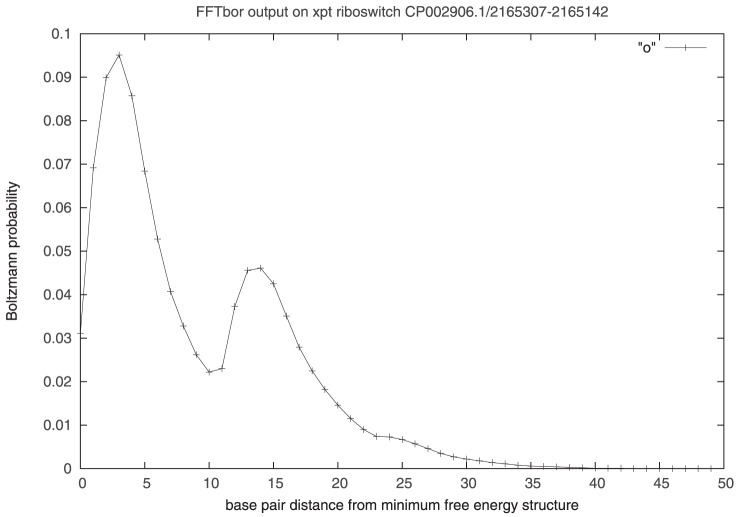
The 161 nt xpt G-box purine riboswitch described in **Figure 1A** **of** [**27**] **, found on the complement strand of GenBank accession number CP002906.1/c2165302-2165142 in complete genome of** ***B. subtilis subsp. subtilis RO-NN-1*** **.** We extended this 161 nt sequence to a sequence of length 200 nt, by appending flanking downstream genomic nucleotides. The web site http://bioinformatics.bc.edu/clotelab/FFTbor displays a movie of all prefixes of the resulting 200 nt sequence, where prefix lengths range from 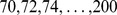.

If the biologically functional target structure is unknown, one can instead attempt a similar moving window computation, where the target structure is taken to be the minimum free energy structure of the current window contents. In this case, one may hope to determine a bimodal distribution, as displayed in Figure 7. Given an input RNA sequence, or genomic region, the web server http://bioinformatics.bc.edu/clotelab/FFTbor creates a movie as follows, described here for the xpt riboswitch previously discussed. We extended the 161 nt xpt G-box purine riboswitch described in Figure 5, with GenBank accession number CP002906.1/c2165302-2165142, to a sequence of length 200 nt, by appending flanking downstream genomic nucleotides. Running FFTbor on all prefixes of the resulting sequence of lengths 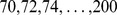, we produced a movie, displayed on the webserver http://bioinformatics.bc.edu/clotelab/FFTbor. Figure 5 displays the output of FFTbor on the 166 nt prefix, clearly showing a bimodal distribution. Attempting to automate the identification of non-unimodal FFTbor output, we have applied the Hartigan-Hartigan dip-test [24], implemented in R; however, the dip-test appears to be too sensitive, in that a probability distribution is reported to be non-unimodal, even when visual inspection indicates that it appears overwhelmingly to be unimodal (data not shown). It is for this reason that the web server http://bioinformatics.bc.edu/clotelab/FFTbor produces a movie of prefixes, where the user can start/stop the movie, move forward/backward, or download all raw data output by FFTbor.

### Benchmarking results

#### Total variation distance for density of states

Recall that the *total variation distance* between two probability distributions 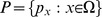 and 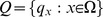, defined on the same sample space 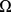, is defined by
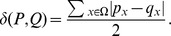
The *density of states* for an RNA sequence 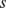 with respect to an initial structure 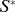 of 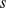 is defined to be the probability distribution 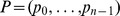 where 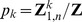. In all our tests, for RNA of length up to 400 nt, we found the total variation distance between 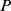, as computed to 6 decimal places by RNAbor and by FFTbor, to be 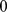. It follows that FFTbor can reliably be used in place of RNAbor to determine Boltzmann probabilities 
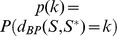
.

#### Run time comparison of RNAbor and FFTbor

**Figure 8:**
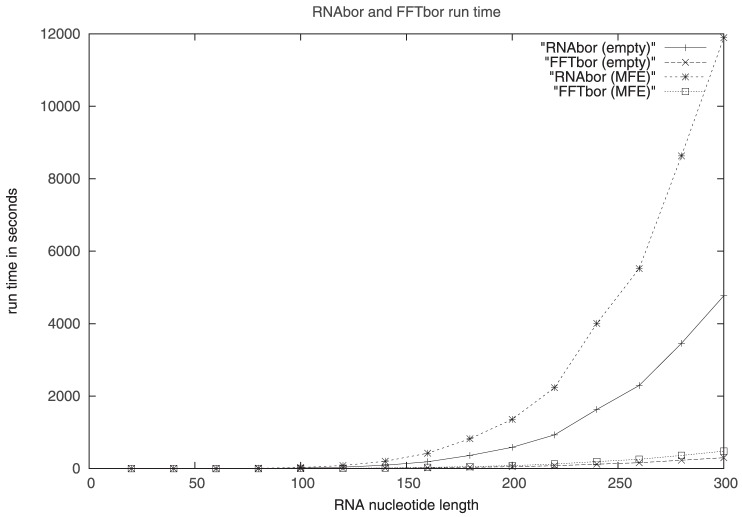
Run times in seconds for RNAbor and FFTbor, on random RNA of length 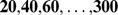
 **in step size of 20 nt. Each algorithm was run with the empty initial structure** 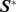 **, see rows RNAbor (empty), FFTbor (empty), and with the minimum free energy structure as the initial structure** 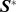 **, see rows RNAbor (MFE) and FFTbor (MFE).** Note that for both RNAbor and FFTbor, the run time increases when 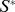 is the MFE structure, rather than the empty structure. Notice the radical improvement in the run time of FFTbor over that of RNAbor.

As visible from the defining recursions, the algorithmic time complexity of RNAbor is 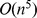 and space complexity is 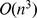, where 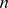 is the length of input RNA sequence. In contrast, the time complexity of FFTbor is 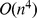 and space complexity is 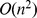. Figure 8 displays run time curves for both RNAbor and FFTbor, when the initial structure 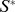 is taken to be either the empty structure or the minimum free energy (MFE) structure.

Here, we compare the run time of RNAbor [1] and the (unparallelized version of) FFTbor, using a Dell Power Edge 1950, 2× Intel Xeon E5430 Quad core with 2.80 GHz and 16 GB RAM. For 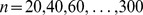, in step size of 20 nt, we generated 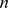 random RNA sequences of length 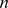 with equal probability for each nucleotide A,C,G,U (i.e. a 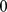
th order Markov chain). For values of 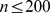, 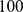 random sequences of length 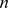 were generated, while for values of 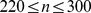, only 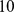 sequences of length 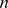 were generated. RNA sequences larger than 300 nt were not tested, due to 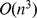 memory constraints required by RNAbor. For each RNA sequence, RNAbor and FFTbor were both run, each starting with empty initial structure 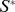, and also with initial sequence 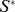 taken to be the MFE structure. Each data point in the table comprises the average run time for three independent evaluations.

#### OpenMP parallelization of FFTbor

**Figure 9:**
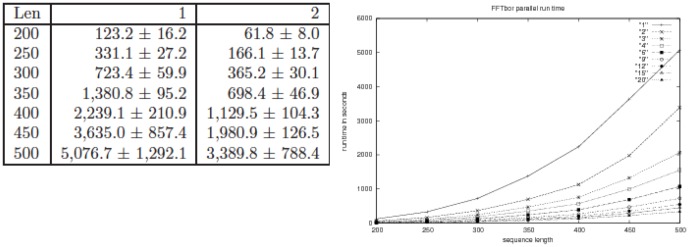
*(Left)* Table showing parallel run times in seconds for FFTbor, using OpenMP http://openmp.org/ **.** Column headers 1,2 indicate the number of cores used in the computational experiment. For each sequence length 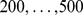, five random RNAs were generated using equal probability for each nucleotide A,C,G,U. Run time in seconds, plus or minus one standard deviation, are given for a 24-core AMD Opteron 6172 with 2.10 GHz and 64 GB RAM, with only 1 (resp. 2) cores used. *(Right)* Graph showing parallel run time of FFTbor on an AMD Opteron 6172 with 2.10 GHz and 64 GB RAM, using respectively 1,2,3,4,6,9,12,15,20 cores.

OpenMP is a simple and flexible multi-platform shared-memory parallel programming environment, that supports parallelizations of C/C++ code – see http://openmp.org/. Using OpenMP primitives, we created multiple threads to evaluate the polynomial 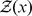 on different complex 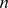
th roots of unity. The table in the left panel of Figure 9 and Table 2 together present benchmarks, executed on a 24-core AMD Opteron 6172 with 2.10 GHz and 64 GB RAM, for the speedup of FFTbor as a function of the number of cores. The table in Figure 9 describes average run time in seconds (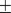 one standard deviation) for running FFTbor on random RNA of length 200,250,300,400,450,500 with either 1 or 2 cores. Table 2 presents similar data for running FFTbor on 2,3,6,4,12,15,20 cores. Although FFTbor clearly has quartic 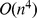 run time as a function of RNA sequence length, least-squares fit of run times from Table 2 instead shows a quadratic run time for RNA sequences of length up to 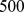 nt. There appears to be a *power law* dependence of FFTbor speedup, as a function of number of cores. For instance, for random RNA of length 200 nt, least-squares fit of the data from the table yields a run time of 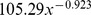 with 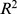 value of 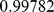. A power law behavior is demonstrated, with similarly high 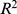 values, for each fixed sequence length in Table 2, with different coefficients of variable 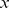 but with approximately the same exponent of 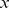 (data not shown, but easily computable from data in Table 2).

**Table 2:** Table showing parallel run times of FFTbor, using OpenMP http://openmp.org/. Column headers 2,3, etc. indicate the number of cores used in the computational experiment.

Len	2	3	4	6	9	12	15	20
200	61.8 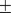 8.0	41.6 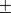 6.0	31.6 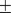 4.2	21.1 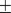 2.9	15.0 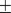 2.3	11.3 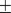 1.1	9.6 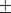 1.3	7.6 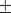 1.5
250	166.1 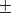 13.7	111.5 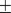 8.7	84.1 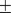 7.1	56.9 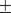 4.0	38.8 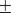 3.8	30.3 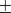 2.6	24.6 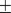 1.6	18.9 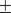 2.7
300	365.2 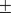 30.1	246.4 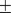 20.5	184.5 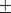 14.9	125.1 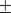 9.6	85.5 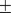 6.6	64.9 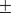 6.5	53.6 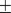 5.9	42.1 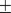 5.2
350	698.4 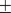 46.9	470.1 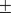 32.6	352.0 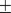 23.0	242.6 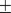 15.8	163.3 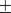 12.4	125.1 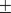 6.7	104.2 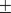 8.9	76.2 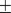 3.9
400	1,129.5 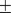 104.3	757.5 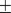 68.9	571.6 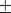 53.9	391.2 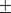 36.4	265.1 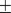 24.2	207.2 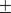 18.4	165.6 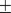 14.5	125.9 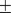 14.9
450	1,980.9 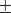 126.5	1,326.3 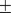 85.1	1,000.0 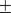 59.0	688.9 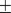 44.8	469.2 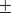 29.1	355.1 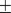 25.4	289.8 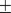 21.2	223.1 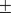 18.2
500	3,389.8 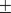 788.4	2,067.9 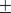 99.2	1,555.0 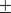 72.2	1,074.3 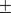 53.7	728.1 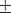 40.9	548.3 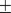 24.0	451.5 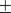 25.7	338.1 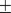 22.7

For each sequence length 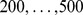, five random RNAs were generated using equal probability for each nucleotide A,C,G,U. Run time in seconds, plus or minus one standard deviation, are given for a 24-core AMD Opteron 6172 with 2.10 GHz and 64 GB RAM. Least-squares fit of the data indicates a quadratic dependency of run time on sequence length (despite the obvious 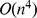 theoretical run time), and a power law dependence of approximately 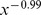 on the number of cores 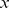.

## Conclusion and Discussion

In this paper, we have used a dynamic programming computation to evaluate the polynomial
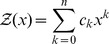
(22)on the complex 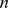
th roots of unity 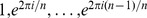, where the coefficients 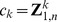 are equal to the sum of Boltzmann factors over all secondary structures of a given RNA sequence, whose base pair distance to a given initial structure 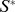 is 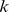. Recall the definition of polynomial
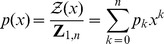
(23)obtained from 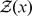, whose coefficients are Boltzmann probabilities 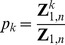 that a secondary structure has base pair distance 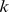 to 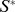. By using the fast Fourier transform to compute the inverse discrete transform, we can approximate to 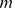 decimal places the coefficients 
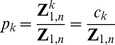
 of 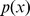, and thus the 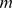 most significant positions of 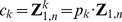. Interpolation is performed for 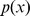, rather than 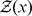, due to issues concerning numerical instability. The computational advantage of FFTbor over its predecessor RNAbor [1] is that the new algorithm runs in quartic time 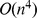 and quadratic space 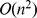, in contrast to the 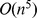 run time and 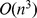 space required by RNAbor. We have additionally provided a parallelization of FFTbor using OpenMP primitives. Additionally, we have described applications of FFTbor to determine the correlation between kinetic folding speed and the *ruggedness* of the energy landscape, and to predict the location of riboswitch expression platform candidates.

It is important to point out that the algorithm and software RNAbor is more general than that of FFTbor – in particular, RNAbor not only computes the partition function values 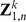, for all 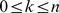, but as well as computes the structures 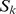, defined to be the minimum free energy structure over all 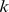-neighbors of initial structure 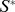. In contrast, FFTbor only computes the 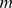 most significant digits of the probabilities 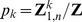, for 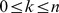, where by multiplication of 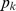 by the partition function 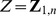, one obtains an approximation of the partition function values 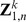. There is no possibility that FFTbor can compute the structures 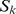, nor can at present we see how to use FFTbor to sample structures from the Boltzmann ensemble of structures having base pair distance 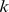 from 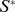.

In [37], [38], we introduced the a related *parametric* RNA structure algorithm, RNAmutants, which computes the partition function 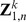 and minimum free energy structure 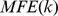 over all secondary structures of all 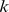-point mutants of a given RNA sequence 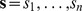. In [39], RNAmutants was extended to sample low energy structures over 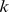-point mutants within a certain range of GC-content. Some of the ideas in [39] foreshadowed the results of this paper, and in the future, we intent to apply interpolation and the FFT to similarly provide a more efficient version of RNAmutants. Nevertheless, this future, more efficient version will be incapable of efficiently sampling low energy structures over 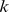-point mutants, analogous to the current differences between RNAbor and FFTbor.

## Supporting Information

File S1 **Supplementary information.**
(PDF)
