# Supplementary material for: Using the Fast Fourier Transform to Accelerate the Computational Search for RNA Conformational Switches
Source: PLoS One. 2012 Dec 19;7(12):e50506. doi: 10.1371/journal.pone.0050506 (PMC3526635; doi:10.1371/journal.pone.0050506)
Supplement: File S1 — Supplementary information. (PDF) [file pone.0050506.s001.pdf]

Supporting Information: Using the Fast Fourier Transform to accelerate the computational search for RNA conformational switches

Evan Senter[1], Saad Sheikh[2], Ivan Dotu[1], Yann Ponty[3], Peter Clote[1*]

1: Biology Department, Boston College, Chestnut Hill, MA, USA.
2: Computer Science Department, University of Florida, Gainesville, Florida, USA.
3: Laboratoire d'Informatique, Ecole Polytechnique, Palaiseau, France.

Full recursions for $\mathcal{Z}_{i,j}(x)$ for the Turner energy model

In the main paper, the recursions given in Theorem 1 and related material in the section Methods are for the simple Nussinov energy model for RNA secondary structure, in which base pairs are assigned a stabilizing energy of -1. This was done to simplify the argument on first reading. Nevertheless, it is known that the Turner energy function, which considers stabilizing energy contributions due to base stacking and destabilizing energy contributions due to loop entropy, is much more accurate for structure prediction. For that reason, our software, `FFTbor`, is implemented for the Turner energy model, following the following recursions.

To compute $\mathcal{Z}(x) = \mathbf{Z}_{1,n}(x)$, we use the recursions

$$\mathbf{Z}_{i,j}(x) = \mathbf{Z}_{i,j-1}(x) \cdot x^{d_0} + \sum_{\substack{s_k s_j \in \mathbb{B}, \\ i \le k < j}} \Big(\mathbf{Z}_{i,k-1}(x) \cdot \mathbf{ZB}_{k,j}(x) \cdot e^{-E_d/RT} \cdot x^{d_1} \Big), \tag{1}$$

where $d_0 = 1$ if j is base paired in $S_{[i,j]}$ and 0 otherwise, where $d_1 = d_{BP}(S_{[i,j]}, S_{[i,k-1]} \cup S_{[k,j]})$, and where E_d is the energy contribution due to dangling ends (energy contributions from single bases stacking on adjacent base pairs) and closing AU base pairs (since a non GC base pair closing a stem has a destabilizing effect). The sum is taken over all possible base pairs (k,j) with $i \le k < j$.

We compute $\mathbf{ZB}(x)$ using the recursion

$$\begin{aligned} \mathbf{ZB}_{i,j}(x) &= e^{-EH(i,j)/RT} \cdot x^{d_2} \\ &+ \sum_{\substack{s_k s_l \in \mathbb{B}, \\ i<k<l<j}} \mathbf{ZB}_{k,l}(x) \cdot e^{-EI(i,j,k,l)/RT} \cdot x^{d_3} \\ &+ \sum_{\substack{s_k s_l \in \mathbb{B}, \\ i<k<l<j}} \Big(\mathbf{ZM}_{i+1,k-1}(x) \cdot \mathbf{ZB}_{k,l}(x) \cdot e^{-(a+b+c(j-l-1))/RT} \cdot x^{d_4} \Big), \end{aligned} \tag{2}$$

where $d_2 = d_{BP}(S_{[i,j]}, \{(i,j)\})$, where $EH(i,j)$ is the energy of the hairpin loop with closing base pair (i,j), $EI(i,j,k,l)$ is the energy of the stack, bulge or interior loop with the closing base pair (i,j) and the interior base pair (k,l), $d_3 = d_{BP}(S_{[i,j]}, S_{[k,l]} \cup \{(i,j)\})$, and $d_4 = d_{BP}(S_{[i,j]}, S_{[i+1,k-1]} \cup S_{[k,l]} \cup \{(i,j)\})$. The first term in the recursion takes care of the case where (i,j) is the only base pair in $[i,j]$, i.e. (i,j) closes a hairpin loop. The second term handles the case where there is an interior loop (or a bulge or a stack) closed by (i,j) and (k,l). The third term takes care of all the structures where (i,j) closes a multi-loop. To reduce

*Corresponding author, `clote@bc.edu`. First and last authors should be considered joint first authors.

complexity of the algorithm, the interior and bulge loop size can be limited to a maximum size of L, by requiring that $l > j - L$ in the above recursion.

The final recursion, for computing $\mathbf{ZM}(x)$, is

$$\begin{aligned}\mathbf{ZM}_{i,j}(x) &= \mathbf{ZM}_{i,j-1}(x) \cdot e^{-c/RT} \cdot x^{d_0} \\ &+ \sum_{\substack{s_k s_j \in \mathbb{B}, \\ i \le k < j}} \Big(\quad \mathbf{ZB}_{k,j}(x) \cdot e^{-(b+c(k-i))/RT} \cdot x^{d_5} \\ &\qquad + \mathbf{ZM}_{i,k-1}(x) \cdot \mathbf{ZB}_{k,j}(x) \cdot e^{-b/RT} \cdot x^{d_6} \Big),\end{aligned} \tag{3}$$

where $d_5 = d_{BP}(S_{[i,j]}, S_{[k,j]})$ and $d_6 = d_{BP}(S_{[i,j]}, S_{[i,k-1]} \cup S_{[k,j]})$. Note that since $\mathbf{ZM}_{i,j}(x)$ computes the partition function contribution under the assumption that $[i,j]$ is part of a multi-loop, there will be exactly one stem-loop structure in this region (the $\mathbf{ZB}(x)$ term) or more than one (the $\mathbf{ZB}(x) - \mathbf{ZM}(x)$ term). Justification of recursions (1), (2), and (3) follow by induction, as in the proof of Theorem 1.

Scaling

Since the use of scaling may not be well-known in the context for RNA secondary structure, we describe how the recursions of `FFTbor` can be scaled to any given constant. Let $c > 1$ be a real scaling constant. Given an RNA sequence $a = a_1, \ldots, a_n$ and initial structure S_0 of a, let $\mathbf{Q}^k_{1,n} = \frac{\mathbf{Z}^k_{1,n}}{c^n}$ denote the *scaled* sum of Boltzmann factors of all secondary structures S, whose base pair distance from S_0 is exactly k. Noting that the maximum base pair distance between any two structures of a is at most n, we define the *polynomial*

$$Q(x) = \sum_{k=0}^{n} c_k x^k \tag{4}$$

whose coefficients $c_k = \mathbf{Q}^k_{1,n}$. If we evaluate the polynomial $Q(x)$ for $n+1$ distinct values

$$Q(x_1) = y_1, \ldots, Q(x_{n+1}) = y_{n+1}$$

then the Lagrange interpolation formula guarantees that $Q(x) = \sum_{k=1}^{n} y_i \cdot P_k(x)$, where

$$P_k(x) = \frac{\prod_{i \ne k}(x - x_i)}{\prod_{i \ne k}(x_k - x_i)}.$$

Let $\mathbf{Q}(x)$ denote $\mathbf{Q}_{1,n}(x)$, defined by induction on $j - i$ as follows. For $1 \le i \le j \le i + \theta$, define $\mathbf{Q}_{i,j} = \frac{1}{c^{j-i}}$, while for $i + \theta + 1 \le j \le n$,

$$\mathbf{Q}_{i,j}(x) = \frac{1}{c} \cdot \mathbf{Q}_{i,j-1}(x) \cdot x^{d_0} + \sum_{\substack{s_k\ s_j \in \mathbb{B}, \\ i \le k < j}} \Big(\frac{1}{c} \cdot \mathbf{Q}_{i,k-1}(x) \cdot \mathbf{QB}_{k,j}(x) \cdot e^{-E_d/RT} \cdot x^{d_1} \Big), \tag{5}$$

where $d_0 = 1$ if j is base paired in $\mathcal{S}_{[i,j]}$ and 0 otherwise, where $d_1 = d_{BP}(\mathcal{S}_{[i,j]}, \mathcal{S}_{[i,k-1]} \cup \mathcal{S}_{[k,j]})$, and where E_d is the energy contribution due to dangling ends (energy contributions from single bases stacking on adjacent base pairs) and closing AU base pairs (since a non GC base pair closing a stem has a destabilizing effect). The sum is taken over all possible base pairs (k, j) with $i \le k < j$.

Let $\mathbf{QB}(x)$ denote $\mathbf{QB}_{1,n}(x)$, defined by induction on $j - i$. For $1 \le i \le j \le i + \theta$, define $\mathbf{QB}_{i,j}(x) = 0$, while for $i + \theta + 1 \le j \le n$,

$$\begin{aligned}\mathbf{QB}_{i,j}(x) &= \frac{1}{c^{j-i}} \cdot e^{-EH(i,j)/RT} \cdot x^{d_2} \\ &+ \sum_{\substack{s_k s_\ell \in \mathbb{B}, \\ i<k<\ell<j}} \frac{1}{c^{(j-\ell)+(k-i)}} \mathbf{QB}_{k,\ell}(x) \cdot e^{-EI(i,j,k,\ell)/RT} \cdot x^{d_3} \\ &+ \sum_{\substack{s_k s_\ell \in \mathbb{B}, \\ i<k<\ell<j}} \Big(\frac{1}{c^{j-\ell+2}} \cdot \mathbf{QM}_{i+1,k-1}(x) \cdot \mathbf{QB}_{k,\ell}(x) \cdot e^{-(a+b+c(j-\ell-1))/RT} \cdot x^{d_4} \Big),\end{aligned} \tag{6}$$

where $d_2 = d_{BP}(\mathcal{S}_{[i,j]}, \{(i,j)\})$, where $EH(i,j)$ is the energy of the hairpin loop with closing base pair (i,j), $EI(i,j,k,\ell)$ is the energy of the stack, bulge or interior loop with the closing base pair (i,j) and the interior base pair (k,ℓ), $d_3 = d_{BP}(\mathcal{S}_{[i,j]}, \mathcal{S}_{[k,\ell]} \cup \{(i,j)\})$, and $d_4 = d_{BP}(\mathcal{S}_{[i,j]}, \mathcal{S}_{[i+1,k-1]} \cup \mathcal{S}_{[k,\ell]} \cup \{(i,j)\})$. The first term in the recursion takes care of the case where (i,j) is the only base pair in $[i,j]$, i.e. (i,j) closes a hairpin loop. The second term handles the case where there is an interior loop (or a bulge or a stack) closed by (i,j) and (k,ℓ). The third term takes care of all the structures where (i,j) closes a multi-loop. To reduce complexity of the algorithm, the interior and bulge loop size can be limited to a maximum size of L, by requiring that $l > j - L$ in the above recursion.

Let $\mathbf{QM}(x)$ denote $\mathbf{QM}_{1,n}(x)$, defined as follows. For $1 \leq i \leq j \leq i+\theta$, define $\mathbf{QM}_{i,j}(x) = 0$, while for $j \leq i+\theta+1 \leq n$,

$$\begin{aligned}\mathbf{QM}_{i,j}(x) = &\frac{1}{c} \cdot \mathbf{QM}_{i,j-1}(x) \cdot e^{-c/RT} \cdot x^{d_0} \\ &+ \sum_{\substack{s_k s_j \in \mathbb{B}, \\ i \leq k < j}} \Big(\quad \frac{1}{c^{k-i}} \cdot \mathbf{QB}_{k,j}(x) \cdot e^{-(b+c(k-i))/RT} \cdot x^{d_5} \\ &\qquad\qquad + \frac{1}{c} \cdot \mathbf{QM}_{i,k-1}(x) \cdot \mathbf{QB}_{k,j}(x) \cdot e^{-b/RT} \cdot x^{d_6} \Big),\end{aligned} \tag{7}$$

where $d_5 = d_{BP}(\mathcal{S}_{[i,j]}, \mathcal{S}_{[k,j]})$ and $d_6 = d_{BP}(\mathcal{S}_{[i,j]}, \mathcal{S}_{[i,k-1]} \cup \mathcal{S}_{[k,j]})$. Note that since $\mathbf{QM}_{i,j}(x)$ computes the partition function contribution under the assumption that $[i,j]$ is part of a multi-loop, there will be exactly one stem-loop structure in this region (the $\mathbf{QB}(x)$ term) or more than one (the $\mathbf{QB}(x) - \mathbf{QM}(x)$ term).
